# Life and Death

**Published:** 1870-01

**Authors:** 


### LIFE AND DEATH.

Life is chemical operation, Death is the absence of it. Every breath of the blessed out-door air drawn into our lungs is full freighted with oxygen; this oxygen comes in all its purity and plenteousness from the sun. In every ray of sunlight there are several colors; one of these is yellow, and through its operation on the world about us oxygen is derived.

When the breath above named is returned from the lungs, it is loaded with a deadly product, called carbonic acid gas. The present state of our knowledge seems to show that while human life is supported by oxygen taken into the lungs, vegetable life is sustained and kept in growth by absorbing into itself, and then condensing into a solid substance more or less of this carbonic acid gas which is rejected by man; hence in the economy of a beneficent and wise providence, nothing is lost; hence the wasteful are wicked in not following the example of their great Creator.

Both carbon and oxygen are necessary to the life of animals and plants, but man receives his carbon through the food he eats; it is this which keeps him warm—his oxygen through the lungs. Plants receive both their carbon and oxygen through their lungs, which are their leaves.

If a plant is kept in a cellar or other place where it gets no sunshine, it grows weak, watery and pale, and never comes to maturity.

If a man is deprived of sunlight he fades away like a flower without water, and always comes to an untimely grave.

